# Trends in Treatment Provision for Mental Illness and Substance Use in the United States:1997-2024

**DOI:** 10.1093/geroni/igaf122.387

**Published:** 2025-12-31

**Authors:** Marilyn Sinkewicz, Philippa Clarke, Iris Gomez-Lopez

**Affiliations:** University of Michigan, Ann Arbor, Michigan, United States; University of Michigan, Ann Arbor, Michigan, United States; University of Michigan, Ann Arbor, Michigan, United States

## Abstract

Economic and health shocks, such as the COVID-19 pandemic and the opioid crisis, have intensified concerns about mental health. Recently, the Surgeon General declared a public mental health emergency. For an increasingly aging population the adequate provision of mental health and substance use (MHSU) treatment is important to successfully aging in place. However, evidence to guide treatment provision has largely been limited to specialty services, select geographic areas, and discrete time periods. To inform MHSU planning, policy, and programs for older adult populations, this study uses national longitudinal neighborhood-level data from a wide range of public sources to examine the evolution of treatment provision from 1997 through 2024. We used geospatial hot spot analysis and heat maps to examine overall patterns and trends, as well as nuanced dynamics by source of treatment, demography, and geography. Findings reveal that MHSU treatment is delivered by a wide variety of providers and facilities, and that the overall density of treatment provision increases over time. Yet the distribution of treatment provision is uneven at the turn of the 21th century, and differences widen over the next 24 years. Source-specific trends differ with respect to their trajectories, geographic impact, and demographic groups affected. For example, urban neighborhoods widen their advantage over rural areas; economically affluent neighborhoods improve more than disadvantaged ones; and white neighborhoods progress more than black and Hispanic neighborhoods. The exception is psychiatric hospitals, which become concentrated in disadvantaged rural and black rural areas. Findings suggest tailoring mental health interventions by geographic/demographic area.

